# Suicide in mental health patients in the UK between 2005 and 2021: study of methods and clinical characteristics to inform prevention

**DOI:** 10.1192/bjo.2024.822

**Published:** 2024-12-05

**Authors:** Pauline Rivart, Lana Bojanić, Pauline Turnbull, Louis Appleby, Nav Kapur, Isabelle M. Hunt

**Affiliations:** National Confidential Inquiry into Suicide and Safety in Mental Health (NCISH), Centre for Mental Health and Safety, Faculty of Biology, Medicine and Health, University of Manchester, Manchester, UK; NIHR Greater Manchester Patient Safety Research Collaboration, University of Manchester, Manchester, UK; Mersey Care NHS Foundation Trust, Prescot, UK

**Keywords:** Suicide, methods, mental health patients, clinical management, prevention

## Abstract

**Background:**

Tackling methods of suicide and limiting access to lethal means remain priority areas of suicide prevention strategies. Although mental health services are a key setting for suicide prevention, no recent studies have explored methods used by mental health patients.

**Aims:**

To investigate associations between main suicide methods and social, behavioural and clinical characteristics in patients with mental illness to inform prevention and improve patient safety.

**Method:**

Data were collected as part of the National Confidential Inquiry into Suicide and Safety in Mental Health. We examined the main suicide methods of 26 766 patients in the UK who died within 12 months of contact with mental health services during 2005–2021. Associations between suicide methods and patient characteristics were investigated using chi-square tests and univariate and multivariate logistic regression.

**Results:**

Suicide methods were associated with particular patient characteristics: hanging was associated with a short illness history, recent self-harm and depression; self-poisoning with substance misuse, personality disorder and previous self-harm; and both jumping and drowning with ethnic minority groups, schizophrenia and in-patient status.

**Conclusions:**

A method-specific focus may contribute to suicide prevention in clinical settings. Hanging deaths outside of wards may be difficult to prevent but our study suggests patients with recent self-harm or in the early stages of their illness may be more at risk. Patients with complex clinical histories at risk of suicide by self-poisoning may benefit from integrated treatment with substance use services. Environmental control initiatives are likely to be most effective for those at risk of jumping or drowning.

A key objective within the national suicide prevention strategies of UK countries includes reducing access to means of suicide, either by restricting availability or by making means less lethal.^1–4^ The most recent strategy for England highlights the need to continue addressing means and methods of suicide, with an emphasis on reducing access and limiting awareness of methods, to provide opportunities for intervention.^2^ Similarly, the National Institute for Health and Care Excellence (NICE) quality standards for suicide prevention recommend agencies use local data to identify trends in suicide methods and frequently used locations.^5^

Multiple factors are associated with suicide methods, including physical availability, that is, the ease of access to a particular method,^6^ and cognitive availability, that is, the awareness and acceptability of a suicide method, which is typically influenced by media portrayals, culture and personal experience of self-harm.^7,8^ However, there is also evidence that individuals or groups with specific demographic, clinical or behavioural features may be more likely to select a particular method over others.^9^ Importantly for prevention, in some cases, a suicide attempt may be delayed or even prevented if a preferred method is unavailable, effectively providing time to intervene.^10^

Much research has examined common suicide methods in the general population and helped provide broad prevention strategies through a public health approach.^11^ Less is known about suicide methods of patients under the care of mental health services. Increasing knowledge of common factors associated with particular methods could aid clinicians to intervene, and potentially reduce mental health patient suicides. Evidence from national and international studies has shown differing clinical characteristics of people with mental illness who die by various suicide methods. These include associations between affective disorder and hanging;^9,12^ psychosis and jumping or drowning, possibly because of heightened impulsivity;^9,12–14^ in-patients and jumping or drowning, a group with limited access to other physical means;^12^ and substance use disorders and self-poisoning,^9,12^ which may also be linked to ease of access.

Much of this research relates to data collected over a decade ago and there is a need for more recent evidence. Further knowledge of suicide method by people with mental illness may provide opportunities for services to address these risk factors and assess and manage suicide risk accordingly.^9^ The aims of this study were to describe the sociodemographic, behavioural and clinical characteristics of people under mental healthcare in the UK who died by each of the most common suicide methods between 2005 and 2021 to make recommendations for clinical practice to improve safety for all patients.

## Method

### Data collection

Data were collected as part of the National Confidential Inquiry into Suicide and Safety in Mental Health (NCISH). The NCISH methodology is described fully elsewhere^15^ but in brief, data collection involves three stages. First, we obtained information on all deaths in the UK that received a suicide or undetermined conclusion at inquest. These data were provided by the Office for National Statistics (ONS) in England and Wales, the National Records of Scotland (NRS) and the Northern Ireland Statistics and Research Agency (NISRA). We included deaths receiving a conclusion of undetermined intent as is conventional in suicide research in the UK, and to avoid underestimation of suicide cases.^16^ These are referred to as ‘suicides’ in this paper. Second, people who had been in contact with mental health services within 12 months of death were identified via administrative contacts in each mental health service provider. The 12-month criterion was chosen as patients recently seen by services may be more amenable to preventative efforts. Third, clinical data were collected via questionnaires completed by the patient's supervising clinician. The questionnaire included sections on sociodemographic characteristics, psychosocial history, details of the suicide (including method), clinical history and aspects of care.

Details on method of suicide in the general population were obtained from the ONS, NRS and NISRA. For the patient population, the method was obtained from the clinician who selected from the following list: hanging/strangulation; self-poisoning (including the substances used); jumping (including jumping from a height and jumping/lying in front of a moving vehicle); drowning; gas inhalation; burning; cutting/stabbing; suffocation/asphyxiation; electrocution; firearms; and other specified methods. In cases where the method was not known, this was determined either from the ONS, NRS or NISRA data. The sample described here consists of all people who died by suicide in the UK between January 2005 and December 2021 inclusive. A previous NCISH study^9^ examined method of suicide in mental health patients between 2000 and 2004, and we therefore included patients who died from 2005 onwards.

Ethical approval was obtained from the North West Research Ethical Committee (reference: ERP/96/136). As patient identifiable data were collected for medical research without patient consent, NCISH has Section 251 approval under the National Health Service (NHS) Act 2006 (reference: 23/CAG/0024).

### Statistical analysis

The methods of suicide selected for analysis were hanging/strangulation, self-poisoning, jumping, drowning, cutting/stabbing and gas inhalation. These were the most common methods in the UK between 2005 and 2021, both in the general population and in the patient population, accounting for 97% of all patient suicide deaths.

Patient variables included in the study were selected based on previous literature on associations between characteristics and methods of suicide. Multicollinearity was checked for all independent variables using variance inflation factors (VIFs), and these ranged from 1.08 to 5.11 (mean VIF = 1.92).

Descriptive results are presented as frequencies and proportions, with denominators based on valid cases for the item as missing data were excluded from the analyses. Chi-square tests were used for subgroup analyses. Univariate analyses were conducted using logistic regression to identify associations between individual variables and suicide method. These are presented as odds ratios with 99% confidence intervals. Risk factors for each method were then identified by multivariate analyses. Variables included in the multivariate logistic regression models were those found to be significantly associated with each specific method in the univariate analyses for ease of interpretation. We ran complete (Model A) and backwards stepwise (Model B) multivariate logistic regressions. Goodness of fit, using likelihood-ratio tests, was greater in complete models; however, backwards stepwise models provided more parsimonious models with similar odds ratios, and we therefore report odds ratios and 99% confidence intervals from the full models and backwards stepwise models. We also examined variables for males and females separately.

To control for multiple comparisons, the significance of all univariate and multivariate analyses was determined using Bonferroni-adjusted alpha levels of 0.0017 (α = 0.05/number of comparisons). While the current study is exploratory, we opted for a conservative alpha level to avoid Type I error. Analyses were conducted using Stata 16 software for Windows.

## Results

**Fig. 1:**
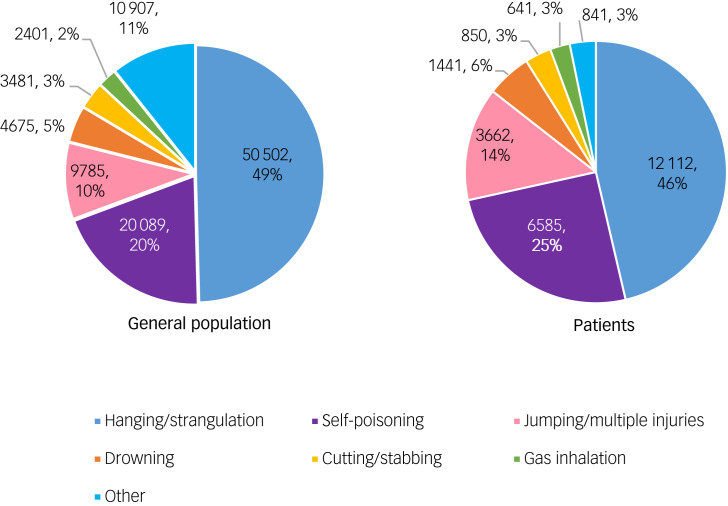
Methods of suicide in the general population and in patients with mental illness in the UK between 2005 and 2021.

Between 1 January 2005 and 31 December 2021, there were 102 163 deaths by suicide in the general population. Of these, 26 766 (26.2%) had been in contact with mental health services in the 12 months before death. Figure 1 shows the methods of suicide in the general population, the most common being hanging/strangulation, self-poisoning and jumping. Other methods included firearms (*n* = 3257, 3.2%), burning (*n* = 1181, 1.2%), suffocation/asphyxiation (*n* = 1094, 1.1%), electrocution (*n* = 222, 0.2%) and other specified (*n* = 4769, 4.0%). Method of suicide was unknown for 202 (0.2%) individuals.

Of the 26 766 patients who died by suicide, 17 595 (65.7%) were male and 9171 (34.3%) were female, a male to female ratio of 2:1. The median age was 45 (interquartile range: 35–56). As with the general population, the most common methods of suicide among patients were hanging/strangulation, self-poisoning and jumping (Fig. 1). Other methods included suffocation/asphyxiation (*n* = 603, 2.3%), burning (*n* = 351, 1.4%), firearms (*n* = 128, 0.5%), electrocution (*n* = 57, 0.2%) and other specified (*n* = 365, 1.4%). The method was unknown for 123 (0.5%) patients. Compared to suicides in the general population, patients had a higher proportion of deaths by self-poisoning, jumping, drowning and cutting/stabbing, but a lower proportion were by hanging/strangulation and gas inhalation.

The most common drugs used by patients who died by self-poisoning were opioids (*n* = 2039, 35%), antidepressants (*n* = 1052, 18%), antipsychotics (*n* = 619, 11%) and paracetamol (*n* = 379, 6%). Of those who died using opioids and where this information was available (*n* = 981), these had been prescribed to the patient in 46% (*n* = 450) of cases.

Hanging/strangulation, self-poisoning and jumping were the most common methods for men and women, although men were significantly more likely to die by hanging/strangulation and women by self-poisoning (see Table 1).

**Table 1:** Methods of suicide in those who died by suicide within 12 months of contact with mental health services by gender

	Male *N* = 17 595		Female *N* = 9171		
Method	*n*	*%*	*n*	*%*	*P-*value
Hanging/strangulation	8717	49.7	3401	37.2	<0.001
Self-poisoning	3473	19.8	3118	34.1	<0.001
Jumping^a^	2461	14.0	1201	13.1	0.04
Drowning	839	4.8	603	6.6	<0.001
Cutting/stabbing	679	3.9	171	1.9	<0.001
Gas inhalation	507	2.9	134	1.5	<0.001
Other^b^	532	3.0	309	3.4	0.12

a. Including jumping from a height and jumping/lying in front of a moving vehicle.

b. Including firearms, burning, electrocution, suffocation and other specified.

### Characteristics of patients who died by the most common suicide methods

The frequency and proportions of patient characteristics by main suicide method are presented in Supplementary Tables 1 and 2 available at https://doi.org/10.1192/bjo.2024.822. The univariate and multivariate results of factors associated with each suicide method, adjusted by age and gender, are shown in Supplementary Table 3 and Table 2 (of the main text), respectively. Variables associated with each suicide method compared to all other methods of suicide are summarised below.

**Table 2:** Effect of sociodemographic characteristics, primary diagnosis, clinical features and behaviour characteristics on suicide method – multivariate results using logistic regression

	Hanging		Self-poisoning		Jumping	
	Model A χ^2^(19) = 963.47**	Model B χ^2^(9) = 942.91**	Model A χ^2^(29) = 1630.05**	Model B χ^2^(14) = 1573.27**	Model A χ^2^(21) = 657.92**	Model B χ^2^(12) = 627.82**
Characteristic	Odds ratio [99% CI]	Odds ratio [99% CI]	Odds ratio [99% CI]	Odds ratio [99% CI]	Odds ratio [99% CI]	Odds ratio [99% CI]
Constant	2.94 [2.33–3.72]**	2.22 [1.97–2.50]**	0.05 [0.04–0.07]**	0.07 [0.06–0.09]**	0.19 [0.15–0.24]**	0.14 [0.12–0.17]**
**Sociodemographic**						
Age group						
<25	Ref	Ref	Ref	Ref	Ref	Ref
25–44	0.81 [0.69–0.95]**	−	1.38 [1.10–1.72]**	−	0.82 [0.67–1.00]	
45–64	0.72 [0.60–0.85]**	0.86 [0.79–0.94]**	1.52 [1.20–1.92]**	−	0.72 [0.58–0.88]**	0.85 [0.76–0.96]**
65+	0.48 [0.39–0.58]**	0.60 [0.53–0.68]**	1.53 [1.25–1.88]**	−	0.65 [0.51-0.83]**	0.78 [0.65-0.93]**
Female	0.59 [0.54–0.65]**	0.60 [0.55–0.65]**	2.19 [1.97–2.44]**	2.17 [1.95–2.40]**	−	−
Ethnic minority group	−	−	0.71 [0.57–0.88]**	0.68 [0.55–0.85]**	1.42 [1.17–1.71]**	1.46 [1.21–1.79]**
Unmarried	0.90 [0.81–1.01]	−	1.11 [0.96–1.29]	−	−	−
Unemployed	0.92 [0.84–1.01]	−	1.11 [0.991–1.24]	−	−	−
Living alone	0.82 [0.74–0.91]**	0.76 [0.70–0.82]**	1.41 [1.25–1.60]**	1.51 [1.37–1.67]**	0.92 [0.83–1.03]	−
Homeless	−	−	0.69 [0.47–1.01]	−	−	−
**Clinical**						
Schizophrenia	0.65 [0.58–0.74]**	0.65 [0.58–0.73]**	0.87 [0.74–1.03]	−	1.89 [1.58–2.25]**	1.93 [1.68–2.20]**
Bipolar disorder	−	−	−	−	1.24 [1.00–1.54]*	1.27 [1.05–1.53]**
Depressive disorder	1.17 [1.06–1.28]**	1.18 [1.08–1.30]**	0.99 [0.86–1.13]	−	0.94 [0.80–1.10]	−
Alcohol dependence/misuse	−	−	0.98 [0.78–1.22]	−	0.87 [0.65–1.17]	−
Drug dependence/misuse	−	−	1.36 [1.04–1.78]*	1.61 [1.28–2.03]**	0.85 [0.60–1.21]	−
Personality disorder	0.98 [0.85–1.13]	−	1.30 [1.09–1.56]**	1.33 [1.14–1.56]**	0.73 [0.57–0.93]**	0.76 [0.61–0.94]**
Comorbid psychiatric disorder	0.98 [0.98–1.07]	−	1.18 [1.06–1.32]**	1.21 [1.08–1.34]**	−	−
Short history of illness (<12 months)	1.24 [1.10–1.39]**	1.23 [1.10–1.38]**	0.64 [0.54–0.76]**	0.65 [0.55–0.77]**	−	−
Long history of illness (>5 years)	0.78 [0.70–0.86]**	0.75 [0.68-0.82]**	1.35 [1.19–1.54]**	1.40 [1.24–1.58]**	−	−
In-patient at time of death	−	−	−	−	1.75 [1.46–2.09]**	1.79 [1.50–2.14]**
Died within 3 months of discharge	−	−	−	−	1.34 [1.16–1.55]**	1.34 [1.16–1.54]**
Missed last appointment	−	−	0.99 [0.87–1.11]	−	0.88 [0.76–1.02]	−
Non-adherence with medication	−	−	0.75 [0.65–0.88]**	0.75 [0.64–0.87]**	1.25 [1.07–1.46]**	1.23 [1.05–1.43]**
Recent contact (<1 week)	−	−	0.71 [0.64–0.79]**	0.70 [0.63–0.78]**	1.42 [1.26–1.60]**	1.43 [1.27–1.62]**
**Behavioural**						
History of self-harm	−	−	1.26 [1.12–1.43]**	1.25 [1.10–1.45]**	0.83 [0.74–0.93]**	0.83 [0.74–0.93]**
Recent (<3 months) self-harm	1.28 [1.17–1.40]**	1.26 [1.15–1.37]**	0.75 [0.66–0.85]**	0.74 [0.65–0.84]**	−	−
History of alcohol misuse	0.89 [0.79–1.01]	−	1.38 [1.18–1.60]**	1.32 [1.19–1.48]**	0.80 [0.67-0.95]**	0.77 [0.69-0.87]**
Recent (<3 months) alcohol misuse	1.08 [0.95–1.23]	−	0.88 [0.75–1.03]	−	1.07 [0.88–1.30]	−
History of drug misuse	1.08 [0.95–1.23]	−	1.11 [0.95–1.31]	−	0.92 [0.77–1.11]	−
Recent (<3 months) drug misuse	0.98 [0.85–1.14]	−	1.13 [0.94–1.35]	−	0.95 [0.77–1.16]	−
(*Continued*)						

	Drowning		Cutting/stabbing		Gas inhalation	
	Model A χ^2^(20) = 472.91**	Model B χ^2^(8) = 443.89**	Model A χ^2^(9) = 246.08**	Model B χ^2^(7) = 232.94**	Model A χ^2^(15) = 173.36**	Model B χ^2^(5) = 148.63**
Characteristic	Odds ratio [99% CI]	Odds ratio [99% CI]	Odds ratio [99% CI]	Odds ratio [99% CI]	Odds ratio [99% CI]	Odds ratio [99% CI]
Constant	0.02 [0.01−0.02]**	0.02 [0.01−0.03]**	0.02 [0.01−0.03]**	0.02 [0.01−0.03]**	0.14 [0.09−0.22]**	0.10 [0.07−0.16]**
**Sociodemographic**						
Age group						
<25	Ref	Ref	Ref	Ref	Ref	Ref
25–44	1.20 [0.77–1.87]		2.59 [1.32–2.09]**	2.71 [1.38–5.31]**	−	−
45–64	2.11 [1.36–3.27]**	1.81 [1.47–2.24]**	4.25 [2.18–8.28]**	4.38 [2.25–8.52]**	−	−
65+	4.99 [3.15–7.91]**	4.06 [3.20–5.16]**	5.66 [2.83–11.33]**	5.69 [2.86–11.33]**	−	−
Female	1.47 [1.24–1.75]**	1.41 [1.19–1.67]**	0.50 [0.39−0.64]**	0.50 [0.39−0.63]**	0.44 [0.32−0.59]**	0.45 [0.34−0.61]**
Ethnic minority group	1.36 [1.00–1.86]*	−	−	−	−	−
Unemployed	1.17 [0.97–1.42]	−	−	−	0.88 [0.68–1.15]	−
Living alone	−	−	1.23 [1.00–1.51]*	−	−	−
**Clinical**						
Schizophrenia	1.76 [1.38–2.25]**	1.96 [1.60–2.40]**	1.89 [1.47–2.43]**	2.10 [1.67–2.66]**	0.33 [0.20-0.55]**	0.32 [0.20-0.50]**
Depressive disorder	0.96 [0.78–1.18]	−	0.81 [0.63–1.03]	−	0.90 [0.67–1.20]	−
Alcohol dependence/misuse	1.15 [0.79–1.68]	−	−	−	0.45 [0.24-0.83]**	0.47 [0.27-0.84]**
Drug dependence/misuse	0.86 [0.46–1.60]	−	−	−	0.53 [0.24–1.14]	−
Personality disorder	0.77 [0.52–1.13]	−	−	−	1.43 [0.96–2.16]	−
Comorbid psychiatric disorder	0.77 [0.65−0.92]**	0.77 [0.64−0.91]**	−	−	0.69 [0.53−0.89]**	0.68 [0.54−0.87]**
Short history of illness (<12 months)	−	−	1.41 [1.11–1.80]**	1.34 [1.05–1.69]**	−	−
Long history of illness (>5 years)	−	−	−	−	0.91 [0.70–1.17]	−
In-patient at time of death	1.78 [1.37–2.32]**	1.64 [1.27–2.12]**	−	−	0.40 [0.19–0.82]**	0.38 [0.19–0.76]**
Died within 3 months of discharge	1.19 [0.96–1.49]	−	−	−	0.85 [0.60–1.20]	-
Recent contact (<1 week)	−	−	1.36 [1.11–1.67]**	1.34 [1.09–1.64]**	0.83 [0.64–1.07]	−
**Behavioural**						
History of self-harm	0.89 [0.73–1.08]	−	−	−	−	−
Recent (<3 months) self-harm	0.92 [0.73–1.14]	−	−	−	−	−
History of alcohol misuse	1.11 [0.84–1.46]	−	−	−	1.06 [0.80–1.41]	−
Recent (<3 months) alcohol misuse	1.19 [0.88–1.61]	1.32 [1.07–1.62]**	−	−	−	−
History of drug misuse	0.68 [0.49–0.93]*	0.71 [0.56–0.89]**	−	−	0.91 [0.61–1.35]	−
Recent (<3 months) drug misuse	1.08 [0.73–1.58]	−	−	−	0.99 [0.62–1.58]	−

*P < 0.01, **P < 0.0017.

Model A = full multivariate logistic regression model; Model B = backwards stepwise multivariate logistic regression model; variables with a *P*-value greater than 0.0017 are removed.

### Hanging/strangulation

The variables, adjusted by age and gender, associated with suicide by hanging/strangulation were as follows: being aged under 25 (adjusted odds ratio [AOR] = 1.96, 99% CI 1.63–2.35); male gender (AOR = 1.68, 99% CI 1.55–1.83); a diagnosis of depressive disorder (AOR = 1.18, 99% CI 1.08–1.30); recent (<3 months) self-harm (AOR = 1.26, 99% CI 1.15–1.37); and a short (<12 months) history of illness (AOR = 1.23, 99% CI 1.10–1.38).

### Self-poisoning

Factors associated with suicide by self-poisoning were as follows: female gender (AOR = 2.17, 99% CI 1.95–2.40); living alone (AOR = 1.51, 99% CI 1.37–1.67); a primary diagnosis of drug dependence/misuse (AOR = 1.61, 99% CI 1.28–2.03) or personality disorder (AOR = 1.33, 99% CI 1.14–1.56); any comorbid mental health diagnosis (AOR = 1.21, 99% CI 1.08–1.34); a long (>5 years) duration of illness (AOR = 1.40, 99% CI 1.24–1.58); a history of self-harm (AOR = 1.25, 99% CI 1.10–1.45); and a history of alcohol misuse (AOR = 1.32, 99% CI 1.19–1.48).

### Jumping

Factors associated with suicide by jumping were as follows: being aged under 25 (AOR = 1.34, 99% CI 1.11–1.62); ethnic minority group (AOR = 1.46, 99% CI 1.21–1.79); schizophrenia (AOR = 1.93, 99% CI 1.68–2.20); bipolar disorder (AOR = 1.27, 99% CI 1.05–1.53); current in-patient status (AOR = 1.79, 99% CI 1.50–2.14); recent (<3 months) discharge from psychiatric in-patient care (AOR = 1.34, 99% CI 1.16–1.54); non-adherence with medication (AOR = 1.23, 99% CI 1.05–1.43); and recent (<1 week) contact with services (AOR = 1.43, 99% CI 1.27–1.62).

### Drowning

The following factors were associated with suicide by drowning: aged 65 and over (AOR = 4.06, 99% CI 3.20–5.16); female gender (AOR = 1.41, 99% CI 1.19–1.67); schizophrenia (AOR = 1.96, 99% CI 1.60–2.40); being an in-patient (AOR = 1.64, 99% CI 1.27–2.12); and recent alcohol misuse (AOR = 1.32, 99% CI 1.07–1.62).

### Cutting/stabbing

Patients who died by cutting/stabbing were more likely to be aged 65 and over (AOR = 5.69, 99% CI 2.86–11.33), male (AOR = 2.02, 99% CI 1.58–2.57), diagnosed with schizophrenia (AOR = 2.10, 99% CI 1.67–2.66), have a short (<12 months) history of illness (AOR = 1.34, 99% CI 1.05–1.69) and recent (<1 week) contact with services (AOR = 1.34, 99% CI 1.09–1.64).

### Gas inhalation

The only factor associated with suicide by gas inhalation was being male (AOR = 2.21, 99% CI 1.65–2.95). Data on suicide-related internet use were collected since 2012, and where these were available (*n* = 9158), we found that over a third of patients who died by gas inhalation had evidence of suicide-related internet use, including searching for suicide method, significantly more than those who died using other suicide methods (86/226, 38% *v.* 616/8,909, 7%, *P* < 0.001).

### Analysis by gender

When we examined determinants for males and females separately, those associated with hanging/strangulation in males were identical to the full model; in females, the only variables associated with hanging/strangulation were being aged under 25 and recent contact with services.

Living alone, a long (>5 years) history of illness and a history of alcohol misuse were associated with self-poisoning in both males and females; however, self-poisoning in males was also associated with a diagnosis of drug dependence/misuse, a comorbid mental health diagnosis and a history of self-harm. In females, self-poisoning was associated with being aged 65 and over, unmarried, a diagnosis of personality disorder and recent drug misuse.

Jumping was associated with ethnic minority background, a diagnosis of schizophrenia and current in-patient status in both males and females; in addition, males were more likely to have bipolar disorder and to have recently (<3 months) been discharged, while females were more likely to have had recent service contact.

Females and males who died by drowning were more likely to be aged 65 and over, to be diagnosed with schizophrenia and be an in-patient. Drowning was also associated with recent alcohol misuse in males only.

Cutting/stabbing was associated with schizophrenia in both males and females, and with older age and recent last contact in males only. No variables were associated with gas inhalation in either males or females.

## Discussion

### Main findings

This national consecutive-case series study revealed specific characteristics associated with different methods of suicide among people in recent contact with mental health services. Suicide by hanging/strangulation was associated with being male and younger age, depression, a short illness history and recent self-harm. Patients who died by self-poisoning were more often female, with diagnoses of drug dependence/misuse and personality disorder and previous self-harm and alcohol misuse. We also found that opioids were the most commonly used substance and in around half of cases these had been prescribed for the patient. Deaths by jumping were associated with ethnicities other than White British, schizophrenia and bipolar disorder, current in-patient status or recent discharge and non-adherence with medication. Drowning was strongly associated with older age, female patients, schizophrenia and in-patient status. Suicide by cutting/stabbing was also associated with older age and schizophrenia, and with recent service contact and a short history of illness, while deaths by gas inhalation were associated with being male.

### Findings in relation to previous work

In contrast with Huisman et al,^12^ who reported that patients under mental health services diagnosed with a depressive disorder were less selective with methods, we found depressive disorder was only associated with suicide by hanging. Other factors associated with hanging, such as male gender and younger age, are in line with previous research,^9,12^ suggesting that these characteristics are persistent concerns for this method of suicide.

The presence of social adversity, drug and alcohol misuse and diagnoses of personality disorder in those who died by self-poisoning has previously been reported.^9,12^ With respect to the use of opioids in self-poisoning, Oquendo and Volkow^17^ noted that populations likely to receive prescriptions for opioids may also be those at increased risk of suicide, including people with chronic pain or mood disorders. Indeed, in our study 44% of those who died using opioids also had a major comorbid physical illness, including 34% with chronic pain. NICE guidelines on management of chronic pain recommend that those with chronic pain should not be prescribed opioids because of issues around dependence and withdrawal.^18^

Suicide by jumping in mental health patients has previously been identified as more common among people from an ethnic minority background.^9,19^ Recent research on suicide in ethnic minority patients has evidenced that some of these groups may present to services with fewer indicators of suicide risk and more rapidly changing risk, possibly because of differences in expressing mental distress.^19^ This may lead to individuals choosing more impulsive methods of suicide, such as jumping.^12^ Earlier work has also highlighted an association between jumping and the presence of psychotic disorders, with suggestions that impulsivity and rapid changes in mood and cognition linked to active psychotic symptoms (e.g. delusions, hallucinations) may increase the likelihood of selecting more violent methods.^20^ Importantly, ethnic minority background and a diagnosis of schizophrenia, two of the main risk factors for jumping in our study, have been found to be associated with each other.^21^ This could be because of the reported inequalities experienced by ethnic minority groups in accessing mental healthcare and receiving appropriate treatment,^22^ potentially resulting in people with serious mental illness reaching services later and being more severely ill than White patients.

Studies carried out in both general and patient populations have identified women and people aged 65 and above to be at greater risk of suicide by drowning,^9,23^ which is consistent with our findings. In clinical groups, there is some evidence of an association between drowning and schizophrenia^24^ and with being an in-patient.^9,25^ The higher prevalence of drowning among in-patients has been linked to the proximity of rivers near hospitals and lack of access to other means of suicide.^25^

Research on cutting/stabbing is scarce and has mainly been examined in relation to self-harm or attempted suicide. Kimura et al^26^ found that those who attempted suicide by self-stabbing were more often male and older compared to those who attempted suicide by jumping; however, mood disorders were more common and psychotic disorders less common. In contrast, we found those who died by cutting/stabbing were more likely to have schizophrenia, although this was in comparison with patients dying by all other methods. There is also limited evidence on gas inhalation deaths as a whole, with most research focusing on specific gases and case reports.^27^ Nonetheless, in a recent Canadian study, Sinyor et al^28^ identified a heightened risk of dying by gas inhalation in males, as well as a reduced risk of being diagnosed with a psychiatric disorder.

### Clinical implications

This study has identified particular patient factors associated with each of the specific methods of suicide, leading to implications for prevention in clinical practice. Clinicians should be aware of the most common methods of suicide in certain patient groups and be able to explore these when making an assessment.

The prevention of hanging is a major challenge outside of institutional settings. Our finding of higher rates of self-harm in patients who died by hanging may be indicative of escalation in method that clinical staff should be aware of. Importantly, there may be a window of opportunity for services to intervene with those who self-harm and present to the emergency department, and for whom there is an opportunity for secondary prevention through medical management.^29^ In a national cohort study, Runeson et al^30^ reported a high risk of future suicide among people with severe mental illness who had been admitted following a suicide attempt and that the risk was greatest for those who had self-harmed by hanging. Recently, the suicide prevention strategy for England^2^ has emphasised the need for first responders and front-line staff to be equipped to respond to people in crisis. More generally, there may be a place for continuing to challenge perceptions of hanging as a quick, easy, painless and clean method, notably in the media.^31^ Indeed, such views may develop through inappropriate media reporting and providing access to technical details,^10,29,31^ which has led to recommendations for tighter media reporting of suicide.^32^

Patients who died by self-poisoning had commonly presented to services with a range of social, behavioural and clinical issues, and may benefit from joined-up care between services. This should include support from alcohol and drug services to address the high prevalence of substance misuse in this group. Both primary and secondary care, including general practitioners (GPs), pharmacists and mental health services, should ensure appropriate and safe prescribing and close monitoring of access to medication.^33^ This may be particularly relevant for those receiving pharmacological treatment for comorbid physical conditions who are at increased risk of suicide by self-poisoning compared to those with mental illness alone.^34^ Specifically, mental health services should be aware of the risk associated with opioids prescribed for pain and enquire about the availability of these medications at home, especially in older patients.^35^

Environmental control initiatives are likely to be most effective in preventing suicide by jumping and drowning in those with schizophrenia and among in-patients, because of rapid changes in mood, cognition and risk, and ease of access, respectively.^20,25^ While in-patient wards have become safer over time and suicide rates in in-patients have decreased,^36^ in the current study 68% of in-patients died off the ward, including those who died at nearby railway or river locations. Efforts around local planning and training of railway staff as recommended by NICE^5^ are likely to increase the chances of last-minute intervention and may delay impulsive actions. High-frequency locations in close proximity to hospitals and care homes should be considered in suicide prevention strategies, through joint working between clinical commissioners and local authorities.^37^ There is now established evidence that safety measures such as barriers and fences at bridges are effective in preventing suicide.^38^

While gas inhalation was a less common method among patients than in the general population, controlling access to acquiring toxic gases is important to prevention. Limiting access to gases may be challenging, but improving online safety may play an important part in both accessing information on and purchasing these gases.^27^ The Online Safety Act 2023, which was recently passed in the UK, has made positive steps in preventing platforms from posting harmful material relating to suicide and self-harm, and has made encouraging or assisting suicide online a priority offence.

In addition to method-specific recommendations, our findings support consideration of availability to means in clinical practice.^39^ One approach is means-restriction counselling, which aims to educate patients and their family/carer on the risks associated with access to means; this has been found to lead to patients and/or their carers disposing of or improving safe storage of means of suicide present in the home.^40^ Stronger and up-to-date empirical evidence is needed on the effectiveness of means restriction in risk assessment and management, and its implementation in practice.^41^

### Limitations

This was a large UK-wide study using robust case ascertainment and our findings add to the scarce literature on suicide methods among mental health patients. However, it is important to consider certain methodological limitations. First, the observational nature of the study cannot ascertain causality or the exact mechanisms in choosing a specific method. Second, our data relied on the accuracy of patient case notes and judgement from clinicians who were not blind to patient outcome. This may have introduced some bias, although most data variables are based on objective information and the quality of the NCISH methodology has been found to be reliable.^42^ Third, these findings are not generalisable to all individuals with mental illness as our sample only included those in contact with services in the year before death. Fourth, we did not examine less common methods of suicide, such as burning, electrocution and firearms, owing to small numbers but future research may consider investigating these. Finally, we did not investigate whether there was an effect of year on patient characteristics and suicide method. Adjusting for year may be of interest to examine changing trends in risk factors, especially when changes in method selection have been observed in the general population,^43^ and to ensure current practice is informed by recent trends. This is currently being explored in a further study by the research team.

In summary, recognition of features associated with common methods of suicide may help in the development of suicide prevention strategies by providing adequate support to mental health patients. The challenge in reducing suicide by hanging is clear but may be achieved through greater encouragement to seek help during a mental health crisis and effective treatment of depression. The complex clinical profile of those who have died by self-poisoning highlights that preventative efforts should target improved integration across primary and secondary care and dual diagnosis services, as well as safer prescribing. Environmental control initiatives such as barriers and helpline posters are likely to be most effective for those at risk of jumping or drowning. More broadly, responsible media reporting of suicide and engaging patients and their families or carers in conversations on means access and restriction may be beneficial at times of crisis.

## Supporting information

Rivart et al. supplementary material Rivart et al. supplementary material

## Data Availability

All authors had full access to all of the data in the study and take responsibility for the integrity of the data and the accuracy of data analysis; access to the data is currently ongoing. Data from the study cannot be shared owing to information governance restrictions in place to protect confidentiality. Access to data can be requested via application to the Healthcare Quality Improvement Partnership (https://www.hqip.org.uk/national-programmes/accessing-ncapop-data/).
