# Supplementary material for: Suicide in mental health patients in the UK between 2005 and 2021: study of methods and clinical characteristics to inform prevention
Source: BJPsych Open. 2024 Dec 5;10(6):e228. doi: 10.1192/bjo.2024.822 (PMC11698139; doi:10.1192/bjo.2024.822)
Supplement: Rivart et al. supplementary material [file S2056472424008226sup001.docx]

**Supplementary Table 1. Sociodemographic and behavioural characteristics of patients by method of suicide, compared to all other methods, using chi-squares**

|  | Hanging/ strangulation (*N*=12,112) | | | | | Self-poisoning  (*N*=6,585) | | | | | | | | Jumping  (*N*=3,662) | | | | | | | | Drowning  (*N*=1,441) | | | | | | | | Cutting/  stabbing  (*N*=850) | | | | | | | | Gas inhalation  (*N*=641) | | | | | | | All methods  (*N*=26,688) | | | | |
| --- | --- | --- | --- | --- | --- | --- | --- | --- | --- | --- | --- | --- | --- | --- | --- | --- | --- | --- | --- | --- | --- | --- | --- | --- | --- | --- | --- | --- | --- | --- | --- | --- | --- | --- | --- | --- | --- | --- | --- | --- | --- | --- | --- | --- | --- | --- | --- | --- | --- |
| Characteristic | n | | % | | | n | | | | % | | | | n | | | | % | | | | n | | | | % | | | | n | | | | % | | | | n | | | % | | | | n | | | | % |
| **Sociodemographic** |  |  | | | | |  | | | |  | | | |  | | | |  | | | |  | | | |  | | | | |  | | |  | | | |  | | |  | | | | |  | | |
| Age: median (IQR*) | 44 (33-55) | | | | 46 (36-56) | | | | | | | | 44 (33-55) | | | | | | | | 55 (42-67) | | | | | | | | 50 (41-60) | | | | | | | | 44 (34-52) | | | | | | | 45 (35-56) | | | | | |
| Age group |  | | | | | | | |  | | | | | | | |  | | | | | | |  | | | | | | |  | | | | | |  | | | | | | | | |  | | | |
| < 25 | 1,262 | | 10.4 | | | 385 | | | | 5.9 | | | | 397 | | | | 10.8 | | | | 61 | | | | 4.2 | | | | 21 | | | | 2.5 | | | | 48 | | | 7.5 | | | | 2,259 | | | | 6.8 |
| 25-44 | 4,953 | | 40.9 | | | 2,642 | | | | 40.1 | | | | 1,447 | | | | 39.5 | | | | 355 | | | | 24.6 | | | | 267 | | | | 31.4 | | | | 289 | | | 45.1 | | | | 10,409 | | | | 39.0 |
| 45-64 | 4,592 | | 37.9 | | | 2,732 | | | | 41.5 | | | | 1,372 | | | | 37.5 | | | | 607 | | | | 42.1 | | | | 397 | | | | 46.7 | | | | 262 | | | 40.9 | | | | 10,489 | | | | 39.3 |
| ≥ 65 | 1,305 | | 10.8 | | | 826 | | | | 12.5 | | | | 446 | | | | 12.2 | | | | 418 | | | | 29.0 | | | | 165 | | | | 19.4 | | | | 42 | | | 6.6 | | | | 3,531 | | | | 13.2 |
| Female | 3,401 | | 28.1▾ | | | 3,118 | | | | 47.3▴ | | | | 1,201 | | | | 32.8 | | | | 603 | | | | 41.8▴ | | | | 171 | | | | 20.1▾ | | | | 134 | | | 20.9▾ | | | | 9,144 | | | | 20.9 |
| Ethnic minority | 757 | | 6.5 | | | 290 | | | | 4.6▾ | | | | 376 | | | | 10.6▴ | | | | 119 | | | | 8.5 | | | | 45 | | | | 5.5 | | | | 30 | | | 4.8 | | | | 1,720 | | | | 6.7 |
| Unmarried | 7,983 | | 69.8▾ | | | 4,811 | | | | 77.5▴ | | | | 2,527 | | | | 72.1 | | | | 940 | | | | 68.6 | | | | 587 | | | | 72.9 | | | | 455 | | | 74.7 | | | | 18,242 | | | | 72.2 |
| Unemployed | 4,978 | | 44.5▾ | | | 3,169 | | | | 51.6▴ | | | | 1,679 | | | | 48.4 | | | | 546 | | | | 40.1▾ | | | | 353 | | | | 44.2▾ | | | | 229 | | | 38.8▾ | | | | 11,471 | | | | 46.2 |
| Living alone | 4,874 | | 24.9▾ | | | 3,536 | | | | 56.9▴ | | | | 1,586 | | | | 45.2▾ | | | | 666 | | | | 48.5 | | | | 452 | | | | 55.4▴ | | | | 291 | | | 48.3 | | | | 12,024 | | | | 47.7 |
| Homeless | 313 | | 2.7 | | | 114 | | | | 1.8▾ | | | | 99 | | | | 2.8 | | | | 34 | | | | 2.4 | | | | 14 | | | | 1.7 | | | | 13 | | | 2.1 | | | | 611 | | | | 2.4 |
| **Behavioural** |  | | |  | | | |  | | | |  | | | |  | | | |  | | | | |  | | |  | | | | |  | | |  | | | |  | | |  | | | | |  | |
| History of self-harm | 7,416 | | 64.3 | | | 4,482 | | | | 71.1▴ | | | | 2,122 | | | | 60.2▾ | | | | 797 | | | | 58.5▾ | | | | 502 | | | | 62.0 | | | | 421 | | | 68.5 | | | | 16,552 | | | | 64.9 |
| Recent (<3 months) self-harm | 3,635 | | 32.3▴ | | | 1,566 | | | | 26.0▾ | | | | 982 | | | | 28.2 | | | | 323 | | | | 24.2▾ | | | | 223 | | | | 28.2 | | | | 186 | | | 30.7 | | | | 7,228 | | | | 29.2 |
| History of alcohol misuse | 5,484 | | 47.7 | | | 3,446 | | | | 55.0▴ | | | | 1,408 | | | | 40.1▾ | | | | 564 | | | | 4.9▾ | | | | 379 | | | | 47.1 | | | | 258 | | | 42.7 | | | | 12,044 | | | | 47.4 |
| Recent (<3 months) alcohol misuse | 3,470 | | 31.2▴ | | | 2,003 | | | | 33.3▴ | | | | 875 | | | | 25.4▾ | | | | 346 | | | | 25.9▾ | | | | 249 | | | | 31.4 | | | | 167 | | | 27.7 | | | | 7,404 | | | | 30.1 |
| History of drug misuse | 4,296 | | 37.2 | | | 2,616 | | | | 41.7▴ | | | | 1,201 | | | | 34.2 | | | | 318 | | | | 23.2▾ | | | | 292 | | | | 36.0 | | | | 175 | | | 28.9▾ | | | | 9,230 | | | | 36.3 |
| Recent (<3 months) drug misuse | 2,416 | | 21.5▴ | | | 1,427 | | | | 23.5▴ | | | | 668 | | | | 19.4 | | | | 161 | | | | 12.0▾ | | | | 152 | | | | 19.1 | | | | 96 | | | 16.0 | | | | 5,092 | | | | 20.6 |

^a^IQR = interquartile range ▴positively associated (p<.0017)▾negatively associated (p<.0017)

**Supplementary Table 2. Clinical characteristics of patients by method of suicide, compared to all other methods, using chi-squares**

|  | Hanging/ strangulation (*N*=12,112) | | | Self-poisoning  (*N*=6,585) | | | | Jumping  (*N*=3,662) | | | | Drowning  (*N*=1,441) | | | | Cutting/  stabbing  (*N*=850) | | | | Gas inhalation  (*N*=641) | | | | All methods  (*N*=26,688) | | |
| --- | --- | --- | --- | --- | --- | --- | --- | --- | --- | --- | --- | --- | --- | --- | --- | --- | --- | --- | --- | --- | --- | --- | --- | --- | --- | --- |
| Characteristic | n | % | | n | | % | | n | | % | | n | | % | | n | | % | | n | | % | | n | | % |
| **Clinical** |  | |  | |  | |  | |  | |  | |  | |  | |  | |  | |  | |  | |  | |
| Schizophrenia & other psychotic disorders | 1,440 | 12.2▾ | | 915 | | 14.1▾ | | 1,002 | | 27.7▴ | | 308 | | 21.7▴ | | 217 | | 26.1▴ | | 41 | | 6.5▾ | | 4,153 | | 15.9 |
| Bipolar disorder | 870 | 7.8 | | 534 | | 8.8 | | 329 | | 9.7▴ | | 109 | | 8.2 | | 62 | | 7.9 | | 38 | | 6.3 | | 2,025 | | 8.3 |
| Depressive disorder | 3,979 | 35.8▴ | | 1,771 | | 29.3▾ | | 1,011 | | 29.7▾ | | 451 | | 33.9 | | 223 | | 28.3 | | 223 | | 37.1 | | 8,045 | | 32.8 |
| Alcohol dependence/misuse | 1,022 | 8.6 | | 641 | | 9.9▴ | | 201 | | 5.6▾ | | 132 | | 9.3 | | 73 | | 8.8 | | 347 | | 5.9 | | 2,224 | | 8.5 |
| Drug dependence/misuse | 670 | 5.7 | | 471 | | 7.3▴ | | 125 | | 3.5▾ | | 36 | | 2.5▾ | | 25 | | 3.0 | | 19 | | 3.0 | | 1,392 | | 5.3 |
| Personality disorder | 1,109 | 9.4▾ | | 960 | | 14.8▴ | | 248 | | 6.96▾ | | 80 | | 5.7▾ | | 64 | | 7.7 | | 84 | | 13.3 | | 2,653 | | 10.1 |
| Comorbid psychiatric diagnosis | 6,192 | 52.6▾ | | 3,898 | | 60.3▴ | | 1,876 | | 52.1 | | 654 | | 46.3▾ | | 444 | | 53.6 | | 278 | | 44.5▾ | | 13,988 | | 53.7 |
| Short history of illness (<12 months) | 2,744 | 25.5▴ | | 631 | | 10.4▾ | | 694 | | 20.5 | | 275 | | 20.9 | | 197 | | 26.0▴ | | 142 | | 24.7 | | 4,950 | | 20.6 |
| Long history of illness (>5 years) | 5,134 | 47.7▾ | | 4,080 | | 67.5▴ | | 1,831 | | 54.1 | | 741 | | 64.2 | | 446 | | 58.8 | | 275 | | 47.9▾ | | 13,169 | | 54.7 |
| In-patient at time of death | 874 | 7.3 | | 150 | | 2.3▾ | | 457 | | 12.5▴ | | 152 | | 10.6▴ | | 63 | | 7.4 | | 18 | | 2.8▾ | | 1,838 | | 6.9 |
| Died within 3 months of discharge | 1,799 | 15.1 | | 817 | | 12.5▾ | | 668 | | 18.5▴ | | 216 | | 15.2 | | 129 | | 15.4 | | 87 | | 13.8 | | 3,903 | | 14.8 |
| Died within a week of discharge | 271 | 5.84 | | 111 | | 4.47▾ | | 114 | | 6.11 | | 36 | | 5.14 | | 23 | | 7.03 | | 12 | | 5.50 | | 599 | | 5.52 |
| Missed last appointment | 2,631 | 22.4 | | 1,656 | | 25.8▴ | | 648 | | 18.1▾ | | 295 | | 20.9▾ | | 164 | | 19.9 | | 160 | | 25.4▴ | | 5,810 | | 22.3 |
| Non-adherence with medication | 1,388 | 12.8 | | 615 | | 10.7▾ | | 536 | | 15.7▴ | | 165 | | 12.4 | | 120 | | 15.3 | | 73 | | 12.6 | | 3,035 | | 12.7 |
| Recent (<1 week) last contact | 5,629 | 46.9 | | 2,473 | | 38.0▾ | | 2,169 | | 59.6▴ | | 718 | | 50.4 | | 466 | | 55.5▴ | | 249 | | 39.2▾ | | 12,321 | | 46.6 |

▴positively associated (p<.0017)▾negatively associated (p<.0017)

**Supplementary Table 3. Effect of sociodemographic characteristics, primary diagnosis, clinical features and behaviour characteristics on suicide method – univariate results adjusted for sex and age using logistic regression**

|  | Hanging/Strangulation  (*N*=12,112) | | | | Self-poisoning  (*N*=6,585) | | | | Jumping  (*N*=3,662) | | | |
| --- | --- | --- | --- | --- | --- | --- | --- | --- | --- | --- | --- | --- |
| Characteristic | OR | | AOR | | OR | | AOR | | OR | | AOR | |
| **Sociodemographic** |  |  | |  | |  | |  | |  | |  |
| Age group |  | |  | |  | |  | |  | |  | |
| < 25 | Ref | |  | | Ref | |  | | Ref | |  | |
| 25-44 | .72 [.64-.81]** | |  | | 1.65 [1.41-1.93]** | |  | | .76 [.65-.89]** | |  | |
| 45-64 | .61 [.55-.69]** | |  | | 1.71 [1.47-2.00]** | |  | | .71 [.60-.83]** | |  | |
| ≥ 65 | .46 [.40-.53]** | |  | | 1.48 [1.24-1.77]** | |  | | .68 [.56-.82]** | |  | |
| Female | .60 [.56-.64]** | |  | | 2.10 [1.95-2.26]** | |  | | .93 [.84-1.02] | |  | |
| Ethnic minority | .95 [.83-1.08] | | .88 [.77-.1.00] | | .60 [.51-.71]** | | .61 [.51-.72]** | | 1.84 [1.57-2.15]** | | 1.79 [1.52-2.09]** | |
| Unmarried | .80 [.75-.86]** | | .70 [.65-.75]** | | 1.44 [1.32-1.57]** | | 1.59 [1.45-1.74]** | | .996 [.90-1.11] | | .94 [.84-1.05] | |
| Unemployed | .89 [.83-.95]** | | .75 [.70-.81]** | | 1.34 [1.24-1.44]** | | 1.44 [1.33-1.56]** | | 1.11 [1.01-1.22]* | | 1.08 [.97-1.19] | |
| Living alone | .70 [.66-.75]** | | .70 [.66-.75]** | | 1.63 [1.51-1.75]** | | 1.68 [1.55-1.82]** | | .89 [.81-.97]** | | .91 [.82-.997]* | |
| Homeless | 1.28 [1.04-1.58]* | | 1.08 [.87-1.34] | | .69 [.53-.91]** | | .81 [.61-1.06] | | 1.21 [.91-1.61] | | 1.15 [.86-1.54] | |
| **Clinical** |  | |  | |  | |  | |  | |  | |
| Schizophrenia & other psychotic disorders | .59 [.54-.65]** | | .52 [.48-.57]** | | .84 [.75-.93]** | | .90 [.81-1.00] | | 2.36 [2.12-2.63]** | | 2.36 [2.11-2.63]** | |
| Bipolar disorder | .90 [.80-1.02] | | .97 [.86-1.10] | | 1.11 [.96-1.27] | | .99 [.86-1.13] | | 1.23 [1.04-1.44]** | | 1.27 [1.08-1.50]** | |
| Depressive disorder | 1.28 [1.19-1.37]** | | 1.45 [1.35-1.56]** | | .81 [.74-.88]** | | .76 [.70-.83]** | | .85 [.76-.94]** | | .88 [.79-.97]** | |
| Alcohol dependence/misuse | 1.03 [.92-1.16] | | .95 [.85-1.07] | | 1.26 [1.11-1.43]** | | 1.36 [1.19-1.54]** | | .60 [.49-.73]** | | .59 [.48-.72]** | |
| Drug dependence/misuse | 1.13 [.98-1.31] | | .97 [.84-1.12] | | 1.60 [1.37-1.86]** | | 1.82 [1.56-2.12]** | | .60 [.47-.77]** | | .57 [.44-.73]** | |
| Personality disorder | .86 [.76-.95]** | | .87 [.78-.98]* | | 1.85 [1.66-2.07]** | | 1.65 [1.47-1.85]** | | .62 [.52-.74]** | | .59 [.49-.71]** | |
| Comorbid psychiatric disorder | .92 [.87-.990]** | | .89 [.84-.95]** | | 1.43 [1.33-1.54]** | | 1.44 [1.33-1.55]** | | .93 [.85-1.02] | | .92 [.83-1.01] | |
| Short history of illness (<12 months) | 1.72 [1.59-1.87]** | | 1.72 [1.58-1.88]** | | .37 [.33-.42]** | | .39 [.35-.44]** | | .995 [.88-1.12] | | .992 [.88-1.12] | |
| Long history of illness (>5 years) | .60 [.56-.64]** | | .61 [.57-.65]** | | 2.04 [1.88-2.21]** | | 1.92 [1.77-2.09]** | | .97 [.88-1.07] | | 1.00 [.91-1.11] | |
| In-patient at time of death | 1.08 [.95-1.23] | | 1.11 [.97-1.26] | | .25 [.20-.32]** | | .23 [.19-.29]** | | 2.38 [2.04-2.78]** | | 2.39 [2.05-2.79]** | |
| Died within 3 months of discharge | 1.05 [.96-1.15] | | 1.06 [.97-1.17] | | .72 [.64-.80]** | | .69 [.62-.78]** | | 1.50 [1.32-1.70]** | | 1.51 [1.33-1.71]** | |
| Died within a week of discharge | 1.16 [.93-1.44] | | 1.14 [.91-1.42] | | .63 [.48-.83]** | | .65 [.49-.86]** | | 1.29 [.97-1.70] | | 1.29 [.97-1.70] | |
| Missed last appointment | 1.01 [.93-1.09] | | .95 [..88-1.03] | | 1.19 [1.09-1.30]** | | 1.22 [1.11-1.33]** | | .80 [.71-.91]** | | .78 [.70-.89]** | |
| Non-adherence with medication | 1.02 [.92-1.13] | | .98 [.89-1.09] | | .78 [.69-.88]** | | .78 [.69-.89]** | | 1.34 [1.18-1.54]** | | 1.33 [1.16-1.52]** | |
| Recent contact (< one week) | 1.02 [.96-1.09] | | 1.08 [1.01-1.15]* | | .63 [.58-.68]** | | .58 [.54-.63]** | | 1.83 [1.67-2.01]** | | 1.87 [1.71-2.06]** | |
| **Behavioural** |  | |  | |  | |  | |  | |  | |
| History of self-harm | .95 [.89-1.02] | | .96 [.89-1.02] | | 1.46 [1.35-1.59]** | | 1.37 [1.26-1.49]** | | .79 [.72-.87]** | | .77 [.70-.85]** | |
| Recent (<3 months) self-harm | 1.32 [1.23-1.42]** | | 1.33 [1.23-1.43]** | | .81 [.75-.89]** | | .77 [.70-.84]** | | .95 [.85-1.05] | | .93 [.83-1.03] | |
| History of alcohol misuse | 1.02 [.96-1.09] | | .90 [.84-.96]** | | 1.49 [1.39-1.61]** | | 1.69 [1.56-1.83]** | | .71 [.64-.78]** | | .67 [.61-.74]** | |
| Recent (<3 months) alcohol misuse | 1.10 [1.03-1.18]** | | .996 [.92-1.07] | | 1.22 [1.12-1.32]** | | 1.31 [1.21-1.43]** | | .76 [.69-.85]** | | .74 [.66-.82]** | |
| History of drug misuse | 1.07 [1.01-1.15]* | | .86 [.80-.93]** | | 1.35 [1.25-1.46]** | | 1.71 [1.57-1.87]** | | .90 [.82-.993]* | | .81 [.73-.90]** | |
| Recent drug misuse (<3 months) | 1.11 [1.03-1.21]** | | .92 [.84-.998]* | | 1.26 [1.15-1.38]** | | 1.51 [1.37-1.67]** | | .92 [.82-1.04] | | .85 [.75-.96]** | |

* p<.01 **p<.0017; OR = odds ratios; AOR = odds ratios adjusted for sex and age

|  | Drowning  (*N*=1,441) | | | Cutting  (N=850) | | | | Gas inhalation  (*N*=641) | | |
| --- | --- | --- | --- | --- | --- | --- | --- | --- | --- | --- |
| Characteristic | OR | AOR | | OR | | AOR | | OR | | AOR |
| **Sociodemographic** |  | |  | |  | |  | |  | |
| Age group |  |  | |  | |  | |  | |  |
| < 25 | Ref |  | | Ref | |  | | Ref | |  |
| 25-44 | 1.28 [.89-1.83] |  | | 2.80 [1.56-5.04]** | |  | | 1.32 [.88-1.98] | |  |
| 45-64 | 2.21 [1.56-3.14]** |  | | 4.19 [2.35-7.49]** | |  | | 1.18 [.78-1.78] | |  |
| ≥ 65 | 4.84 [3.38-6.93]** |  | | 5.22 [2.86-9.52]** | |  | | .55 [.32-.96]* | |  |
| Female | 1.41 [1.22-1.62]** |  | | .47 [.38-.59]** | |  | | .50 [.39-.64]** | |  |
| Ethnic minority | 1.31 [1.01-1.69]* | 1.61 [1.24-2.09]** | | .81 [.54-1.21] | | .93 [.62-1.39] | | .70 [.43-1.14] | | .67 [.41-1.09] |
| Unmarried | .83 [.71-.97] | 1.04 [.89-1.23] | | 1.04 [.84-1.28] | | 1.20 [.97-1.49] | | 1.14 [.89-1.45] | | 1.04 [.81-1.34] |
| Unemployed | .77 [.66-.89]** | 1.16 [.98-1.36] | | .92 [.76-1.11] | | 1.04 [.85-1.28] | | .73 [.59-.92]** | | .60 [.48-.75]** |
| Living alone | 1.03 [.90-1.19] | .98 [.85-1.14] | | 1.38 [1.14-1.65]** | | 1.25 [1.04-1.51]* | | 1.02 [.83-1.27] | | .99 [.79-1.22] |
| Homeless | 1.02 [.64-1.61] | 1.46 [.91-2.32] | | .70 [.35-1.41] | | .72 [.36-1.46] | | .88 [.43-1.83] | | .73 [.35-1.52] |
| **Clinical** |  |  | |  | |  | |  | |  |
| Schizophrenia & other psychotic disorders | 1.51 [1.27-1.79]** | 1.86 [1.56-2.22]** | | 1.92 [1.56-2.37]** | | 2.02 [1.63-2.49]** | | .36 [.24-.55]** | | .32 [.21-.48]** |
| Bipolar disorder | .99 [.76-1.29] | .94 [.72-1.22] | | .95 [.67-134] | | .97 [.68-1.37] | | .75 [.48-1.15] | | .80 [.51-1.23] |
| Depressive disorder | 1.06 [.91-1.23] | .81 [.69-.95]** | | .80 [.65-.990]* | | .70 [.56-.86]** | | 1.22 [.98-1.52] | | 1.38 [1.10-1.73]** |
| Alcohol dependence/misuse | 1.11 [.87-1.42] | 1.36 [1.06-1.74]** | | 1.04 [.75-1.43] | | .997 [.72-1.38] | | .67 [.43-1.03] | | .58 [.37-.90]** |
| Drug dependence/misuse | .45 [.29-.70]** | .66 [.42-1.03] | | .54 [.32-.92]* | | .60 [.35-1.02] | | .55 [.30-1.00]* | | .46 [.25-.84]** |
| Personality disorder | .52 [.38-.70]** | .62 [.46-.85]** | | .73 [.52-1.03] | | 1.02 [.72-1.44] | | 1.38 [1.01-1.87]* | | 1.53 [1.12-2.10]** |
| Comorbid psychiatric disorder | .73 [.63-.84]** | .82 [.71-.95]** | | .994 [.83-1.19] | | 1.07 [.89-1.29] | | .68 [.56-.84]** | | .65 [.53-.81]** |
| Short history of illness (<12 months) | 1.02 [.85-1.22] | .92 [.77-1.11] | | 1.37 [1.10-1.70]** | | 1.24 [.998-1.55] | | 1.28 [.992-1.65] | | 1.29 [1.00-1.67]* |
| Long history of illness (>5 years) | 1.06 [.92-1.23] | 1.11 [.96-1.29] | | 1.19 [.98-1.44] | | 1.21 [.991-1.47] | | .76 [.61-.94]** | | .73 [.59-.92]** |
| In-patient at time of death | 1.72 [1.35-2.18]** | 1.71 [1.34-2.18]** | | 1.15 [.81-1.64] | | 1.20 [.84-1.70] | | .35 [.18-.67]** | | .36 [.19-.70]** |
| Died within 3 months of discharge | 1.07 [.87-1.32] | 1.07 [.87-1.32] | | 1.08 [.84-1.40] | | 1.09 [.84-1.42] | | .82 [.60-1.13] | | .83 [.60-1.13] |
| Died within a week of discharge | .98 [.62-1.56] | .98 [.62-1.56] | | 1.35 [.76-2.39] | | 1.30 [.73-2.31] | | .89 [.41-1.94] | | .88 [.40-1.90] |
| Missed last appointment | .97 [.78-1.22] | 1.16 [.97-1.39] | | .86 [.68-1.09] | | .92 [.73-1.17] | | 1.12 [.88-1.43] | | 1.04 [.82-1.33] |
| Non-adherence with medication | .98 [.83-1.16] | 1.07 [.86-1.34] | | 1.25 [.96-1.62] | | 1.31 [1.01-1.70]* | | .99 [.72-1.38] | | .95 [.68-1.32] |
| Recent contact (< one week) | 1.17 [1.02-1.35]* | 1.09 [.94-1.25] | | 1.44 [1.20-1.73]** | | 1.45 [1.21-1.74]** | | .73 [.59-.90]** | | .77 [.62-.95]* |
| **Behavioural** |  |  | |  | |  | |  | |  |
| History of self-harm | .75 [.65-.87]** | .85 [.73-.99]* | | .88 [.73-1.06] | | 1.04 [.86-1.26] | | 1.18 [.94-1.48] | | 1.22 [.97-1.53] |
| Recent (<3 months) self-harm | .76 [.64-.90]** | .86 [.72-1.02] | | .95 [.77-1.17] | | 1.10 [.89-1.35] | | 1.08 [.86-1.36] | | 1.11 [.88-1.39] |
| History of alcohol misuse | .76 [.65-.87]** | 1.02 [.87-1.19] | | .990 [.82-1.19] | | 1.02 [.84-1.24] | | .82 [.66-1.02] | | .68 [.55-.85]** |
| Recent (<3 months) alcohol misuse | .80 [.68-.95]** | 1.05 [.89-1.25] | | 1.07 [.88-1.31] | | 1.12 [.91-1.37] | | .89 [.70-1.13] | | .77 [.61-.98]* |
| History of drug misuse | .51 [.41-.58]** | .79 [.65-.95]** | | .990 [.81-1.19] | | 1.20 [.97-1.49] | | .71 [.56-.89]** | | .53 [.41-.68]** |
| Recent (<3 months) drug misuse | .51 [.43-.64** | .80 [.63-1.00] | | .91 [.72-1.15] | | 1.08 [.84-1.39] | | .73 [.55-.97]* | | .59 [.43-.79]** |

* p<.01 **p<.0017; OR = odds ratios; AOR = odds ratios adjusted for sex and age
